# Independent and combined effects of age, body mass index and gestational weight gain on the risk of gestational diabetes mellitus

**DOI:** 10.1038/s41598-020-65251-2

**Published:** 2020-05-22

**Authors:** Heng Yaw Yong, Zalilah Mohd Shariff, Barakatun Nisak Mohd Yusof, Zulida Rejali, Yvonne Yee Siang Tee, Jacques Bindels, Eline M. van der Beek

**Affiliations:** 1 Department of Nutrition and Dietetics, Faculty of Medicine and Health Sciences, Universiti Putra Malaysia, Serdang, Malaysia; 2 Department of Obstetrics and Gynaecology, Faculty of Medicine and Health Sciences, Universiti Putra Malaysia, Serdang, Malaysia; 3 Danone Specialized Nutrition (Malaysia) Sdn Bhd, 59200 Mid Valley City, Lingkaran Syed Putra, Kuala Lumpur Malaysia; 4 Danone Nutricia Research, Uppsalalaan 12, 3584 CT Utrecht, The Netherlands; 5 Department of Pediatrics, University Medical Centre Groningen, University of Groningen, Groningen, The Netherlands

**Keywords:** Risk factors, Metabolic disorders

## Abstract

This study aimed to identify the independent and combined effects of age, BMI at first prenatal visit and GWG on the risk of GDM. A retrospective cohort study of 1,951 pregnant women in Seremban district, Negeri Sembilan, Malaysia. GDM was defined as fasting plasma glucose (FPG) ≥5.6 mmol/l and/or 2-hour postprandial plasma glucose (2hPPG) ≥7.8 mmol/l. A higher percentage of women with GDM had 2 risk factors (29.0%) or >2 risk factors (8.6%) compared to non-GDM women (2 risk factors: 25.5%; >2 risk factors: 5.0%). In general, women with ≥2 risk factors were respectively 1.36–2.06 times more likely to have GDM compared to those without risk factors. Older maternal age and being overweight/obese were significantly associated with risk of GDM. Overweight/obese women with age ≥35 years had 2.45 times higher risk of GDM and having excessive GWG at second trimester further increased the risk of GDM. Age and BMI are independent risk factors for GDM but not GWG in the first and second trimester. The findings emphasize the need to focus on a healthy BMI before pregnancy and optimal GWG during pregnancy to improve pregnancy outcomes.

## Introduction

In 2017, of the 16.2% (21.3 million) live births affected by hyperglycaemia in pregnancy, 86.4% (18.4 million) of these cases were due to gestational diabetes mellitus^1^. Globally, the prevalence of gestational diabetes mellitus (GDM) is estimated at 6–12% of all pregnancies^2^. The National Health and Morbidity Survey (2016) reported that the prevalence of GDM among Malaysian mothers aged 15–49 years old was 13.5%^3^. The GDM rate in Malaysian population (8.7–29.7%)^4–6^ was significantly higher than the reported rates in many Western (2.0–9.2%)^7,8^ and Asian countries (2.8–25.0%)^9–12^.

The known risk factors for GDM are advanced age, obesity, excessive gestational weight gain (GWG), ethnicity, family history of diabetes, history of GDM, high parity, short stature, polycystic ovary syndrome (PCOS) and previous large-for-gestational (LGA) births^13–19^. Advanced maternal age is a well-documented risk factor for GDM, as previous studies consistently reported that older pregnant women were more likely to develop GDM^13,14,20–24^. Although maternal age is an established risk factor, there is no consensus regarding a valid cut-off value. The American Diabetes Association recommended the use of ≥25 years as a risk factor for GDM^25^, but this recommendation is supported by very limited data^26^. A retrospective study of 138,530 Japanese women reported that the prevalence of GDM increased with advancing age, with women aged 40 years and above showing a relative risk of 15.1%^20^.

Studies on the associations between pre-pregnancy BMI and GWG with risk of GDM showed that pre-pregnancy obesity and excessive GWG are independent risk factors for the development of GDM. While overweight or obese women had an increased risk of GDM compared to lean or normal-weight women^27^, excessive GWG, particularly between early and mid-pregnancy, was postively associated with risk of GDM^28,29^. Studies have also reported that overweight or obese women were more likely to show excessive GWG^30–32^ and that pre-pregnancy overweight or obesity combined with excessive GWG confers a higher risk of complications in pregnancy, particularly GDM^15,33^.

The Malaysian National Health and Morbidity Survey (NHMS) showed that in the period between 1996 and 2015, there was a 26.6% increase in the prevalence of overweight and obesity (BMI ≥25.0 kg/m^2^) among females aged ≥18 years old. With the increasing average age of Malaysian women having their first child^34,35^ and the rising prevalence of overweight and obesity among women of childbearing age^36^, the risk of having higher pre-pregnancy BMI and excessive GWG are inevitable^37,38^. Although it is well established that increasing maternal age, overweight/obesity, and excessive GWG are independent determinants of GDM, no study has specifically evaluated the combined effects of maternal age, BMI and GWG on the risk of GDM. Thus, the aim of this study was to identify the independent and combined effects of age, BMI at first prenatal visit and GWG on the risk of GDM.

## Results

### Characteristics of women

The baseline characteristics of 1,951 pregnant women included in the analysis are presented in Table 1. The mean age of women was 29.08 ± 4.44, with about 88.3% of women under35 years. Most of the women were Malays (83.9%), had low-secondary education (59.8%) and were employed (61.0%). About 28.4% of women were nulliparous. The mean height and BMI at first prenatal visit were 1.56 ± 0.06 m, and 24.76 ± 5.49 kg/m^2^, respectively. More than half (54.75%) had a height over 1.56 m. According to the BMI calculated at the first prenatal visit, 556 (28.5%) were overweight, and 292 (15.0%) were obese. Two-thirds of women gained weight above the recommended range in the second trimester (42.9%). The mean fasting plasma glucose (FPG) and 2-hour plasma glucose (2hPG) were 4.37 ± 0.51 mmol/l and 6.10 ± 1.41 mmol/l. About 13.1% of the women were diagnosed with GDM.

**Table 1:** Characteristics of women (N = 1951).

	n (%)	Mean ± SD
Age (years)		29.08 ± 4.44
<35	1723 (88.3)	
≥ 35	228 (11.7)	
**Ethnicity**		
Malay	1636 (83.9)	
Others	315 (16.1)	
**Education**		
Lower-secondary	1166 (59.8)	
Others	785 (40.2)	
**Occupation**		
Housewife	761 (39.0)	
Working	1190 (61.0)	
Parity		1.43 ± 1.31
0	554 (28.4)	
1	521 (26.7)	
2	568 (29.1)	
≥3	308 (15.8)	
Height (m)		1.56 ± 0.06
<1.56	884 (45.3)	
≥1.56	1067 (54.7)	
BMI at first prenatal visit (kg/m^2^)		24.76 ± 5.49
Underweight (<18.50)	207 (10.6)	
Normal (18.50–24.99)	896 (45.9)	
Overweight/ (25.00–29.99)	556 (28.5)	
Obese (≥30.00)	292 (15.0)	
GWG at first trimester (kg)		0.25 ± 0.06
Inadequate (<IOM)	1166 (59.8)	
Adequate (= IOM)	585 (30.0)	
Excessive (>IOM)	200 (10.2)	
GWG at second trimester (kg)		5.90 ± 3.41
Inadequate (<IOM)	626 (32.1)	
Adequate (= IOM)	505 (25.9)	
Excessive (>IOM)	820 (42.0)	
**Maternal glucose level**		
OGTT at 28th weeks of gestation (mmol/L)		
Fasting plasma glucose (FPG)		4.37 ± 0.51
2-hours plasma glucose (2hPG)		6.10 ± 1.41
GDM according to MOH criteria^‡^	255 (13.1)	

Note. ^‡^GDM according to MOH criteria, either of both FPG ≥5.6 mmol/l or 2hPG ≥7.8 mmol/L.

### Risk factors between non-GDM and GDM women

Table 2 shows the presence of risk factors among non-GDM and GDM women. There was a higher percentage of women with GDM having age ≥35 years (19.6%) and being overweight/obese (51.4%) as compared to non-GDM women (10.5% age ≥35 years; 42.3% overweight/ obese). However, no significant difference was observed for GWG in the first and second trimester between non-GDM and GDM women. There was a significant difference in the number of risk factors for GDM between non-GDM and GDM women, in that a higher proportion of GDM women having 2 (29.0%) or more than 2 risk factors (8.6%) compared to non-GDM women (25.5% 2 risk factors and 5.0% >2 risk factors). Only a small proportion of women (0.4–0.8%) had all 4 risk factors.

**Table 2:** Proportion of risk factors between non-GDM and GDM.

Factors	Total (n = 1951)	Non-GDM (n = 1696)	GDM(n = 255)	p-value
		n (%)		
Age ≥35 years	228 (11.7)	178 (10.5)	50 (19.6)	0.001*
Overweight/Obese (≥25.00 kg/m^2^)	848 (43.5)	717 (42.3)	131 (51.4)	0.02*
Excessive GWG at the first trimester	200 (10.3)	177 (10.4)	23 (9.0)	0.49
Excessive GWG at the second trimester	820 (42.0)	713 (42.0)	107 (42.0)	0.36
**Number of risk factors**^**¥**^				
None	582 (29.8)	518 (30.5)	64 (25.1)	0.02*
1 risk factor	756 (38.7)	661 (39.0)	95 (37.3)	
2 risk factors	507 (26.0)	433 (25.5)	74 (29.0)	
>2 risk factors	106 (5.4)	84 (5.0)	22 (8.6)	
3 risk factors	98 (5.0)	78 (4.6)	20 (7.8)	
4 risk factors	8 (0.4)	6 (0.4)	2 (0.8)	

^¥^Age ≥35 years, Overweight/Obese (≥25.00 kg/m^2^), excessive GWG at first trimester, excessive GWG at second trimester.

### The effects of age, BMI and GWG to the GDM risk

Table 3 shows the independent and combined effects of age, BMI at first prenatal visit and GWG at the first and second trimester to the risk of GDM, adjusted for covariates. The findings show that maternal age and BMI at first prenatal visit had significant independent associations with the risk of GDM for both categorical and continuous variable. Women with older age (≥35 years) (aOR = 2.08, 95% CI = 1.42–3.07) were significantly at higher risk for GDM than women aged <35 years. Overweight/obese women (aOR = 1.44, 95% CI = 1.04–1.81) had significantly higher risk for GDM compared to underweight/normal weight women. No significant independent effects were observed between GWG in the first and second trimester with the risk of GDM. Women with two risk factors and >2 risk factors had 1.36 times and 2.06 times higher risk for GDM compared to those women with no risk factor.

**Table 3:** Independent and combine factors associated with the risk of gestational diabetes mellitus (GDM).

	GDM	p-value
	Adjusted OR [95% CI]	
**Factors (continuous)**		
Age (years)	1.06 [1.04–1.10]	0.001*
BMI at first prenatal visit (kg/m^2^)	1.03 [1.01–1.06]	0.01*
GWG at the first trimester (kg)	0.92 [0.85–1.01]	0.05
GWG at second trimester (kg)	0.97 [0.93–1.01]	0.11
**Independent factors (categorical)**		
Age (years)		
<35	1.00	
≥35	2.08 [1.42–3.07]	0.001**
**BMI at first prenatal visit (kg/m**^**2**^**)**		
Underweight (<18.50)	0.78 [0.47–1.31]	0.35
Normal (18.50–24.99)	1.00	
Overweight/obese (≥25.00)	1.44 [1.04–1.81]	0.02*
**GWG at the first trimester (kg)**		
Inadequate (<IOM)	1.25 [0.92–1.70]	0.15
Adequate (= IOM)	1.00	
Excessive (>IOM)	1.01 [0.61–1.66]	0.97
**GWG at the second trimester (kg)**		
Inadequate (<IOM)	1.29 [0.91–1.84]	0.16
Adequate (= IOM)	1.00	
Excessive (>IOM)	1.17 [0.83–1.65]	0.37
**Combined factors**		
Number of risk factors^a^		
No risk factor	1.00	
1 risk factor	1.15 [0.82–1.62]	0.41
2 risk factors	1.36 [1.01–1.96]	0.04*
>2 risk factors	2.06 [1.18–3.58]	0.01*

Note. Non-GDM as the reference group (GDM according to MOH criteria, either of both FPG ≥5.6 mmol/l or 2hPG ≥7.8 mmol/L).

^a^1 risk factor was defined as having any 1 of the 4-risk factors; 2 risk factors was defined as having any 2 of the 4 risk factors; >2 risk factors were defined as having more than 2 risk factors.

Adjusted for ethnicity, parity, gestational weeks at OGTT performed.

*p < 0.05.

Figure 2 provides further insights into the independent and combined effects of significant factors on the risk of GDM. Overweight/obese women had 1.44 times higher risk for GDM while women aged 35 years and above had 2 times higher risk for GDM. Women with both aged 35 years and above and overweight/obese had a higher risk of GDM (aOR = 2.45, 95% CI = 1.50–4.00). The risk for GDM was further increased if the women were aged 35 years and above, overweight/obese and had excessive GWG in the second trimester (aOR = 3.38, 95% CI = 1.83–6.24) compared to women without these risk factors.

## Discussion

The present study showed that within our study population more than two-thirds of women had at least one risk factor, with 38.7% and 31.4% had one risk factor and two or more risk factors. Women aged 35 years and above with BMI of overweight/obese had 2.45 times higher risk for GDM, while women with a combination of three risk factors (aged 35 years and above, overweight/obese and with excessive GWG in the second trimester) had 3.38 times higher risk of GDM compared to women with no risk factors. The clustering of these risk factors might confer a greater risk for adverse outcomes, such as large-for-gestational-age (LGA) infants, caesarean delivery. These findings emphasize the importance of addressing not only the individual established risk factors but also the combined effect of these biological and behavioural factors on risk of GDM.

In the present study, age 35 years and above and overweight/obese were independently associated with the risk of GDM. This finding was consistent with previous studies that reported older pregnant women were more likely to develop GDM^13,14,39^. To date, there has been an increasing trend among women in developing countries to delay their first pregnancy. This trend to postpone childbearing is driven largely by the desire to complete education or professional career development^40^. Thus, the number of women giving birth for the first time at the age of 35 years and above has increased steadily^34^. Similarly, in Malaysia, maternal age for having the first baby is increasing^35^. Older maternal age also increases the risk that women might encounter a second pregnancy soon after the previous delivery, carrying along some additional weight and abdominal fat mass due to a short interpregnancy interval, especially in the case of high GWG in the previous pregnancy and postpartum weight retention. The Malaysian Adult Nutrition Survey (MANS) 2016 showed that about 40–55% of women of reproductive age (20–39 years old) were overweight and obese^36^. As the prevalence of overweight/obesity among women entering pregnancy is likely to increase in Malaysia, this could further contribute to an increase in obesity-related adverse pregnancy outcomes.

Normal ageing is associated with deterioration of endocrine functions, such as the decline in β-cell function and insulin sensitivity^41^. The development of GDM is strongly influenced by an individuals’ reduced ability to secrete insulin. In addition, older women are also at an increased risk for both acute and chronic cardiovascular complications, such as coronary artery disease, atherosclerosis, heart failure, and stroke^42^. The underlying mechanism of how maternal age acts as a risk factor for GDM is still unclear but age related alterations in glucose-insulin regulation as well as vascular aging may contribute to poor pregnancy outcomes in older women.

Overweight/obese women in this study had a significantly higher risk of GDM compared to normal weight women. The findings of the present study are similar to those of previous studies in Western populations^15,17,28,33,43^. Several factors may explain the association between pre-pregnancy BMI and GDM. As metabolic changes occur during pregnancy, particularly declining insulin sensitivity in late pregnancy, obese pregnant women may experience a higher risk of homeostatic dysregulation during pregnancy^44^. Obese women have approximately 40% higher in insulin resistance compared to normal weight women^45^. Inflammation is another possible explanation for the association between obesity and GDM^46^, in that overweight and obesity are associated with increased levels of inflammation. Bastard *et al*. (2000) found that an increase in inflammation, especially interleukin-6 (IL-6), among obese individuals was associated with insulin resistance^47^.

The present study showed that neither GWG in the first nor the second trimester was independently associated with the risk of GDM. This finding was inconsistent with a previous study in China^39^, which reported that GWG in the first and second trimester were associated with the risk of GDM only among obese and aged >35 years women. The inconsistent findings could be due to different methodological differences used, such as study design and timing of measurement. The study in China was a prospective study and weight gain in the first trimester was defined as the difference between weight in the early second trimester and self-reported pre-pregnancy weight. The present study in Malaysia was a retrospective study and the GWG in the first trimester was defined as the difference in body weight between early second trimester and first prenatal visit, likely resulting in a more reliable estimate.

This study also showed that women with a combination of two risk factors (aged 35 years and above & BMI overweight/obese) had a higher risk of GDM as compared to women with individual risk factor of either being 35 years and above or overweight / obese. Although excessive GWG in the second trimester was not a significant risk factor of GDM, the combination of three risk factors (aged 35 years and above, overweight/obese and had an excessive GWG in the second trimester) significantly increased the risk of GDM. This finding indicates that maternal age and BMI are more important risk factors than GWG, and this notion is supported by a recent meta-analysis that found the association between maternal pre-pregnancy BMI with the risk for any adverse outcome, including GDM was stronger than the association of GWG^48^. The absolute risk of overweight women for any adverse outcomes increased from 37.3% for GWG of 2.0–3.9 kg to 56.4% for GWG of 28.0 kg or greater^48^. Since age is a non-modifiable risk factor for GDM, the focus on an effective intervention to prevent the risk of developing of GDM should be aimed at helping overweight or obese women to lose weight before pregnancy and to limit weight gain in pregnancy.

There are several limitations to the present study. As the study population comprised pregnant women from the Seremban district, Negeri Sembilan, the study findings may not be generalizable to all pregnant women in Malaysia. The anthropometric measurements were retrieved from antenatal clinic cards, which could potentially produce information bias and misclassification. This study used the recorded weight and height at first prenatal visit within the first 12 weeks of pregnancy to calculate the early pregnancy BMI which could under- or overestimate GWG. Besides, the IOM recommendations for GWG used for classification of GWG is based on the western population, which may not be appropriate for the current study population, albeit there is currently no recommendation for GWG for Asians available. There were methodological limitations for performing combined effects analysis, as some subgroups were relatively small. Despite these statistical limitations, the study findings may have important public health implications.

## Conclusions

More than two-thirds of women in the present study had at least one risk factor of GDM, with 38.7% with one risk factor and 31.4% had two or more risk factors. Maternal age and early pregnancy BMI were stronger contributors to the risk of GDM than GWG. However, excessive GWG in the second trimester was associated with a further increased risk for GDM, yet only in overweight / obese women aged 35 years and above. With age of having children increasing and more women of reproductive age being overweight or obese, the risk of developing GDM is imminent in Malaysia. There is a need for public health awareness on the importance of having healthy pre-pregnancy BMI, achieving optimal weight gain during pregnancy and retaining minimal postpartum weight, particularly among older age women.

## Methods

### Study design and population

**Figure 1:**
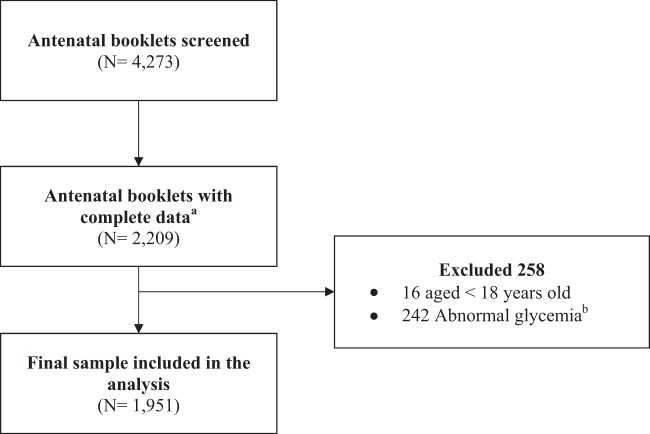
Sampling procedure. Note. ^a^Complete data–complete all antenatal care visits. ^b^Normal glycemia at first prenatal visit was defined as normal plasma glucose for the Ministry of Health Malaysia (MOH) criteria for the diagnosis of GDM^49^.

**Figure 2:**
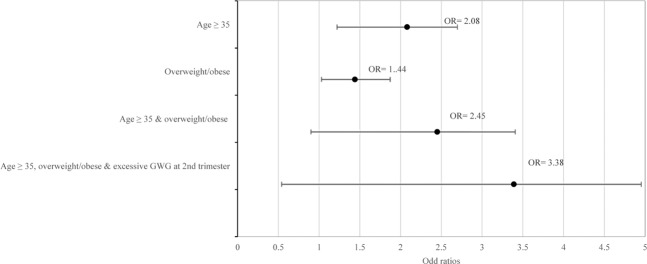
Odd ratios for independent and combined effects of significant factors associated with the risk of GDM. Note. No risk factor as the reference group.

This was a retrospective cohort study of healthy, non-diabetic pregnant women having delivered at government hospitals between January 2010 and December 2012. A total of 4,273 antenatal booklets were screened, and 2,209 pregnant cases were initially identified for potential inclusion into this study. Two hundred and fifty-eight women were subsequently excluded from the analysis as follows: <18 years old (n = 16), and abnormal glycemia (n = 242). The final sample included in the analysis were 1951 pregnant women (Figs. 1 and 2).

The study protocol was approved by the Medical Research Ethics Committee (MREC), Universiti Putra Malaysia (UPM/FPSK/100–9/2-MJKEtika) and the Medical Research Ethics Committee (MREC), Ministry of Health Malaysia (KKM/NIHSEC/08/0804/P12-613), which waived the requirement for informed consent as the data were analysed anonymously. This study was carried out in accordance with Good Clinical Practice (GCP) guidelines and the Declaration of Helsinki.

### Measurements

#### Data sources

The source of data was antenatal clinic cards of pregnant women having delivered at government hospitals between January 2010 and December 2012. The clinic cards contained the patient’s background, antenatal care information, demographic characteristics, and obstetric history. Data were extracted from the antenatal clinic cards by trained enumerators.

#### Maternal glucose level

All pregnant women were required to take a standardized 2-hour 75 g OGTT in between 28th to 32nd week of gestation^49^. GDM was diagnosed if either or both FPG was ≥ 5.6 mmol/l or 2hPG is ≥ 7.8 mmol/l according to the Ministry of Health Malaysia guideline^49^.

#### Anthropometric measurements

Height and weight at first prenatal visit, first trimester, and second trimester were obtained from the antenatal clinic cards. Height and body weight at first prenatal visit were used to calculate early pregnancy Body Mass Index (BMI), as early pregnancy weight (kilogram) divided by the square of height (meter^2^), and further categorized into 4 groups: underweight (<18.5 kg/m^2^), normal weight (18.5–24.9 kg/m^2^), overweight (25.0–29.9 kg/m^2^) and obese (≥30.0 kg/m^2^)^50^. The GWG in the first trimester and second trimester was estimated using the difference between the first and last weight record in that trimester and then classified according to the 2009 US Institute of Medicine (IOM) guidelines, as inadequate, adequate and excessive.

#### Other variables

Information on demographic characteristics and obstetric history were obtained. The variables were classified as follows: age (<35, ≥35 years old), ethnicity (Malay, others), education (lower-secondary, others), occupation (housewife, working), parity (0, 1, 2, ≥3), and height (<1.56, ≥1.56 m).

### Statistical analysis

All analyses were performed using SPPS version 23^51^. Exploratory Data Analysis (EDA) was carried out to determine the normality and homogeneity of the data. All continuous variables were normally distributed. Therefore, no transformation was performed. Basic descriptive statistics were generated such as means and standard deviations for the continuous variables, while for categorical variables, frequency, and percentage distribution.

Binary logistic regression model was used to determine the independent and combined effects of age, BMI at first prenatal visit, GWG in the first and second trimester to the risk of GDM, adjusting for covariates (ethnicity, parity gestational weeks at OGTT performed). Four factors were examined both as continuous and categorical independent variables. Categorical variables included in the analysis were age (<35, ≥35), BMI at first prenatal visit (<18.50, 18.50–24.99, ≥25.00 kg/m^2^), GWG at first and second trimester (inadequate, adequate and excessive), respectively. The combined effects of factors (2 risk factors, 3 risk factors, 4 risk factors) associated with the risk of GDM were further examined using binary logistic regressions. However, the study only reported the significant combined risk factors. Non GDM women served as the reference group. Adjusted odds ratios (ORs) and 95% confidence intervals (CIs) were presented. Significant level for all statistical analysis was set at p < 0.05.

## Data Availability

The datasets used and analyzed during the current study are available from the corresponding author on reasonable request.
